# Quality of vision and vision‐related quality of life after Descemet membrane endothelial keratoplasty: a randomized clinical trial

**DOI:** 10.1111/aos.14741

**Published:** 2021-01-12

**Authors:** Suryan L. Dunker, Mor M. Dickman, Robert P.L. Wisse, Siamak Nobacht, Robert H.J. Wijdh, Marjolijn C. Bartels, N.E. Mei‐Lie Tang, Frank J.H.M. van den Biggelaar, Pieter J. Kruit, Bjorn Winkens, Rudy M.M.A. Nuijts

**Affiliations:** ^1^ Maastricht University Medical Center+ University Eye Clinic Maastricht the Netherlands; ^2^ Department of Ophthalmology University Medical Center Utrecht Utrecht the Netherlands; ^3^ Department of Ophthalmology Radboud University Medical Centre Nijmegen the Netherlands; ^4^ Department of Ophthalmology University Medical Center Groningen Groningen the Netherlands; ^5^ Department of Ophthalmology Deventer Hospital Deventer the Netherlands; ^6^ Department of Ophthalmology Gelre Hospitals Apeldoorn the Netherlands; ^7^ General director ETB‐BISLIFE Leiden the Netherlands; ^8^ Department of Methodology and Statistics Faculty of Health, Medicine and Life Sciences Care and Public Health Research Institute (CAPHRI) Maastricht University Maastricht the Netherlands; ^9^ Department of Ophthalmology Zuyderland Medical Center Heerlen the Netherlands

**Keywords:** corneal transplantation, descemet membrane endothelial keratoplasty, descemet stripping automated endothelial keratoplasty, fuchs endothelial dystrophy, randomized controlled trial

## Abstract

**Purpose:**

To compare quality of vision and vision‐related quality of life (QOL) in patients undergoing Descemet membrane endothelial keratoplasty (DMEK) or ultrathin Descemet stripping automated endothelial keratoplasty (DSAEK).

**Methods:**

Fifty‐four eyes of 54 patients with Fuchs' dystrophy from six corneal clinics in the Netherlands were randomized to DMEK or ultrathin DSAEK and examined preoperatively, and 3, 6 and 12 months postoperatively. Main outcome measures were corneal higher‐order aberrations (HOAs), contrast sensitivity, straylight and vision‐related QOL.

**Results:**

Posterior corneal HOAs decreased after DMEK and increased after ultrathin DSAEK (p ≤ 0.001) 3 months after surgery and correlated positively with best spectacle‐corrected visual acuity (12 months: *r* = 0.29, p = 0.04). Anterior and total corneal HOAs did not differ significantly between both techniques at any time point. Contrast sensitivity was better (p* = *0.01), and straylight was lower (p = 0.01) 3 months after DMEK compared with ultrathin DSAEK; 95% confidence interval [CI] of log(cs) 1.10–1.35 versus 95% CI: 0.84 to 1.12, and 95% CI: log(s) 1.18 to 1.43 versus 95% CI: 1.41 to 1.66, respectively. Both were comparable at later time points. Vision‐related QOL (scale 0–100) did not differ significantly between both groups at any time point and improved significantly at 3 months (*β* = 12 [95% CI: 7 to 16]; p < 0.001), and subsequently between 3 and 12 months (*β* = 5 [95% CI: 0 to 9]; p = 0.06).

**Conclusions:**

Descemet membrane endothelial keratoplasty (DMEK) results in lower posterior corneal HOAs compared with ultrathin DSAEK. Contrast sensitivity and straylight recover faster after DMEK but reach similar levels with both techniques at 1 year. Vision‐related QOL improved significantly after surgery, but did not differ between both techniques.

## Introduction

Fuchs endothelial corneal dystrophy (FECD) is a leading indication for corneal transplantation (Gain et al. 2016). Two transplantation techniques to treat corneal endothelial failure are ultrathin Descemet stripping automated endothelial keratoplasty (ultrathin DSAEK) and Descemet membrane endothelial keratoplasty (DMEK).

We recently reported the results of a multicentre randomized controlled trial (RCT) showing mean best spectacle‐corrected visual acuity (BSCVA) does not differ between DMEK and ultrathin DSAEK, although a significantly higher proportion of patients achieved 0.8 Snellen or better after DMEK (Dunker et al. 2020). Nonetheless, there is more to vision than visual acuity. Patients routinely seek treatment for symptoms related to glare and reduced contrast sensitivity.

In the era of modern endothelial keratoplasty, such complaints may even be the main indication for corneal transplantation in selected cases. The current study provides the most comprehensive evaluation to date of quality of vision and vision‐related quality (QOL) of life after DMEK. In this prespecified analysis, we compare corneal higher‐order aberrations (HOAs), contrast sensitivity, straylight (forward scatter) and vision‐related QOL in patients with symptomatic FECD randomized to either DMEK or ultrathin DSAEK.

## Methods

The full methods of this study were previously reported in detail (Dunker et al. 2020). The current study is a prespecified analysis of a RCT comparing secondary clinical outcomes of DMEK and ultrathin DSAEK over 12 months follow‐up. Patients with corneal dysfunction due to FECD were included in six corneal clinics in the Netherlands. The primary outcome measure was BSCVA in logarithm of minimum angle of resolution (logMAR) using an Early Treatment Diabetic Retinopathy Study (ETDRS) chart 12 months after surgery. The study received approval from the institutional review boards of all participating clinics and complied with the tenets of the Declaration of Helsinki. All participants provided written informed consent. Patients were recruited between November 2016 and November 2017. The trial was registered in the US trial register as the DMEK Versus DSAEK Study (www.clinicaltrials.gov, no. NCT02793310, accessed 15 October 2020).

Inclusion criteria were pseudophakic adult patients with corneal endothelial dysfunction due to FECD. Exclusion criteria were previous corneal transplantation in the study eye, vision‐limiting comorbidities, the need for a human leucocyte antigen‐typed corneal transplantation, or inability to comply with study procedures or complete follow‐up. No triple procedures were performed, and only one eye per patient was enrolled.

### Outcome measures

Patients were evaluated preoperatively, and 3, 6, and 12 months postoperatively. At each visit, corneal HOAs, contrast sensitivity and straylight were measured, and subjects completed the National Eye Institute visual function questionnaire 25 items (NEI VFQ‐25) questionnaire.

Scheimpflug tomography (Pentacam HR, Oculus Optikgeräte GmbH, Wetzlar, Germany) was used to measure corneal HOAs. Measurements were conducted under mesopic conditions using the 50 pictures, 3‐dimensional scan mode with automatic release. All scans were checked for data acquisition errors and repeated if necessary. Corneal aberrations were measured using ray‐tracing over a 6‐mm diameter zone centred at the corneal apex. Using Zernike polynomials, the root mean square (RMS) of the corneal HOAs (3rd–6th order) was calculated.

Contrast sensitivity was measured at 3, 6, 8 and 12 cycles per degree using the CSV‐1000 chart (Vector vision Inc., Greenville, OH, USA). The system's internal light source was calibrated at 85 candelas (cd)/m^2^ and adjusts automatically for ambient light, providing standardized testing. Patients were tested monocularly in undilated eyes at 2.5 m distance with manifest refraction in place. Individual values were converted into the area under the log contrast sensitivity function (log(cs)).

Intraocular forward light scatter (straylight) was measured using the compensation‐comparison based C‐Quant straylight meter (Oculus Optikgeräte GmbH). Refractive error was corrected in the tested undilated eye, while the fellow eye remained occluded. Measurements took place in a dark room. Examinations were considered reliable when estimated standard deviation and quality factor were ≤0.08 and ≥1.00, respectively. Forward light scatter was expressed as the logarithm of the straylight parameter (log(s)).

Vision‐related QOL was assessed using the NEI VFQ‐25 questionnaire. This instrument consists of 11 vision‐related subscales and one scale addressing general health. Each scale ranges from zero to 100, that is from worst to best possible outcome, respectively. A composite score was calculated by averaging all unweighted item scores except general health.

### Sample size

Sample size calculation was based on the primary outcome measure, BSCVA 12 months after surgery. We expected a difference of 0.2 logMAR with a standard deviation of 0.2 logMAR between DMEK and ultrathin DSAEK. Choosing a two‐sided alpha at 5%, a power of 90% and expecting 15% loss to follow‐up, at least 25 subjects were required per treatment arm. Four to five patients were allocated per treatment arm per centre.

### Randomization and blinding

Randomization with minimization was performed centrally by an investigator from the coordinating centre using a random sequence generator (Trans European Network for Clinical Trials Services, TENALEA, available at www.tenalea.net). Patients were randomized based on the following stratification factors: preoperative ETDRS logMAR BSCVA, recipient central corneal thickness, recipient sex, recipient age and recruitment centre. The cornea bank (ETB‐BISLIFE, Leiden, the Netherlands) received the assigned treatment plan and distributed the grafts to surgeons. Patients were blinded throughout the study period. Outcome assessors were unblinded because eyes that underwent DMEK and ultrathin DSAEK are distinguishable during postoperative assessment.

### Statistical analysis

Data were analysed using an intention‐to‐treat analysis. Analyses were performed using spss for Windows (version 24.0, SPSS Inc., Chicago, IL, USA). Categorical data were described as individual counts and percentages and continuous data as mean ± standard deviation. A linear mixed model (LMM) with the respective mean outcome variable as the dependent variable, study group, time and study group*time as factors, and an unstructured covariance matrix was used. Bivariate relationships were calculated using Pearson correlation analysis. Sensitivity analysis was performed for the outcome measure vision‐related quality of life (QOL). Data of patients that underwent corneal transplantation in the fellow eye during the study were excluded at the follow‐up time points 3, 6 and 12 months to eliminate the effect of the fellow eye surgery. A two‐sided p ≤ 0.05 was considered statistically significant.

## Results

**Fig. 1:**
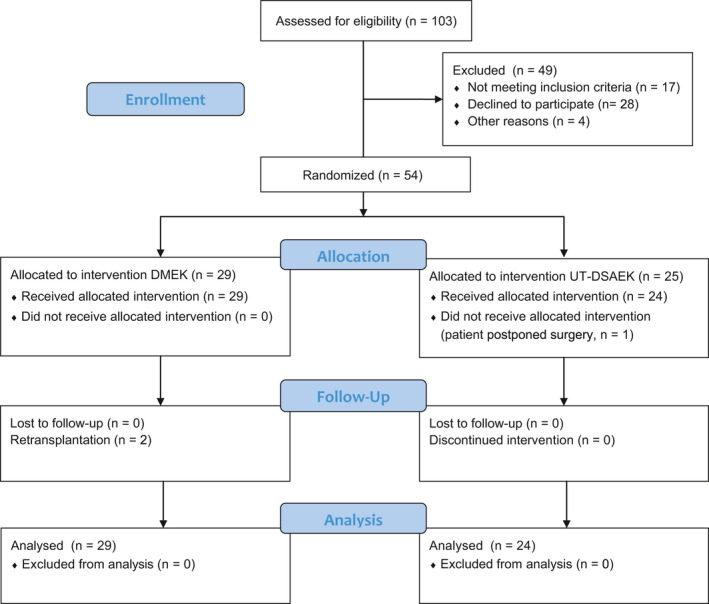
Randomized controlled trial of DMEK versus ultrathin DSAEK: Participant flow diagram. Twenty‐nine eyes of 29 patients were randomized to DMEK, and 25 eyes of 25 patients were randomized to ultrathin DSAEK. One patient in the ultrathin DSAEK arm did not undergo corneal transplantation. DMEK = Descemet membrane endothelial keratoplasty, UT‐DSAEK = ultrathin Descemet stripping automated endothelial keratoplasty.

A study flow diagram is presented in Fig. 1. Briefly, fifty‐four eyes of 54 patients were randomized to DMEK (*n* = 29) or UT‐DSAEK (*n* = 25). All patients in both groups received the allocated treatment, except for one patient in the UT‐DSAEK arm who postponed treatment indefinitely. Two patients in the DMEK arm underwent repeated transplantation due to persistent graft detachment. No patients were lost to follow‐up. In the DMEK arm, patients were 72 [69–74] years old and endothelial cell density of the donor graft measured 2679 [2620–2739] cells/mm^2^. In the ultrathin DSAEK arm, patients were 71 [68–74] years old, endothelial cell density of the donor graft measured 2633 [2567–2700] cells/mm^2^, and preoperative central graft thickness measured 101 ± 25 *µ*m [90–112]. In the DMEK arm, 24% (*n* = 7) underwent cornea transplantation in the other eye before the study, and 31% (*n* = 9) underwent cornea transplantation in the other eye during the study. In the ultrathin DSAEK arm, 21% (*n* = 5) underwent cornea transplantation in the other eye before the study, and 38% (*n* = 9) underwent cornea transplantation in the other eye during the study.

### Corneal higher‐order aberrations

**Fig. 2:**
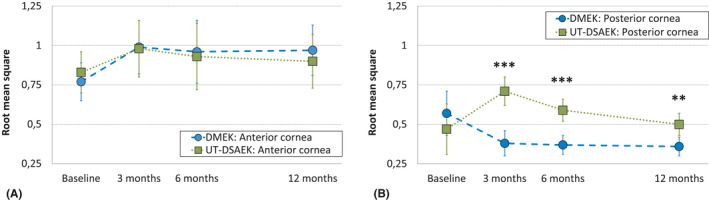
Randomized controlled trial of DMEK versus ultrathin DSAEK: Corneal higher‐order aberrations (HOAs, 3rd to 6th order) over a 6.0‐mm optical zone in both treatment groups. (A) Left: Anterior corneal HOAs did not differ between DMEK and ultrathin DSAEK at all time points. Anterior corneal HOAs increased by *β* = 0.18 [95% CI: 0.01 to 0.34], p = 0.039, and subsequently stabilized between 3 and 12 months. (B) Right: Posterior corneal HOAs were significantly lower after DMEK compared with ultrathin DSAEK at 3 months (rms = 0.38 [95% CI: 0.30 to 0.46] versus rms = 0.71 [95% CI: 0.62 to 0.79]; p < 0.001), 6 months (rms = 0.37 [95% CI: 0.31 to 0.43] versus rms = 0.59 [95% CI: 0.52 to 0.66], p < 0.001) and 12 months (rms = 0.36 [95% CI: 0.30 to 0.41] versus rms = 0.50 [95% CI: 0.43 to 0.56], p = 0.002). Total corneal HOAs (not shown) did not differ significantly between DMEK and ultrathin DSAEK at all time points and did not change significantly over time (all *β* ≤ 0.13, all p ≥ 0.2). DMEK = Descemet membrane endothelial keratoplasty, UT‐DSAEK = ultrathin Descemet stripping automated endothelial keratoplasty. **p ≤ 0.01; ***p ≤ 0.001.

Anterior corneal HOAs did not differ between DMEK and ultrathin DSAEK at all time points, Table 1. Anterior corneal HOAs increased by *β* = 0.18 [95% CI: 0.01 to 0.34], p = 0.039 and subsequently stabilized between 3 and 12 months (*β* = −0.04 [95% CI: −0.2 to 0.13], p = 0.7), Fig. 2A. At 6 months, anterior corneal HOAs significantly correlated with BSCVA (*r* = 0.3, p = 0.033), but not at other time points. Best spectacle‐corrected visual acuity (BSCVA) values are given in Table 1.

**Table 1:** Randomized controlled trial of DMEK versus ultrathin DSAEK: Contrast sensitivity, straylight, corneal higher‐order aberrations and vision‐related quality of life outcomes.

	UT‐DSAEK, EMM [95% CI] (*n*)	DMEK, EMM [95% CI] (*n*)	p
HOAs CF, rms			
Preoperative	0.83 [0.70–0.96] (24)	0.77 [0.65–0.89] (28)	0.5
3 months	0.98 [0.80–1.17] (24)	0.99 [0.82–1.16] (27)	1
6 months	0.93 [0.72–1.14] (23)	0.96 [0.77–1.16] (27)	0.8
12 months	0.90 [0.73–1.08] (24)	0.97 [0.81–1.13] (28)	0.6
HOAs CB, rms			
Preoperative	0.47 [0.31–0.63] (24)	0.57 [0.43–0.72] (28)	0.3
3 months	0.71 [0.62–0.79] (24)	0.38 [0.30–0.46] (27)	**<0.001**
6 months	0.59 [0.52–0.66] (23)	0.37 [0.31–0.43] (27)	**<0.001**
12 months	0.50 [0.43–0.56] (24)	0.36 [0.30–0.41] (28)	**0.002**
HOAs TC, rms			
Preoperative	0.92 [0.71–1.13] (24)	0.95 [0.76–1.15] (28)	0.8
3 months	1.13 [0.93–1.33] (24)	1.04 [0.86–1.23] (27)	0.5
6 months	1.04 [0.81–1.27] (23)	1.00 [0.79–1.22] (27)	0.8
12 months	1.02 [0.84–1.21] (24)	1.02 [0.85–1.19] (28)	1
Contrast sensitivity, log			
Preoperative	0.66 [0.56–0.76] (24)	0.71 [0.61–0.80] (28)	0.5
3 months	0.98 [0.84–1.12] (22)	1.22 [1.10–1.35] (26)	**0.01**
6 months	1.13 [1.03–1.24] (23)	1.23 [1.13–1.33] (28)	0.2
12 months	1.15 [1.03–1.28] (24)	1.27 [1.15–1.39] (28)	0.2
Straylight, log			
Preoperative	1.55 [1.44–1.66] (17)	1.58 [1.47–1.68] (17)	0.7
3 months	1.54 [1.41–1.66] (18)	1.30 [1.18–1.43] (19)	**0.01**
6 months	1.40 [1.30–1.50] (15)	1.35 [1.26–1.45] (18)	0.5
12 months	1.33 [1.23–1.43] (18)	1.34 [1.24–1.45] (15)	0.9
VFQ‐25, composite score			
Preoperative	69 [63–75] (23)	67 [62–72] (27)	0.6
3 months	77 [72–83] (23)	80 [75–85] (29)	0.4
6 months	82 [78–86] (24)	84 [80–88] (28)	0.5
12 months	84 [79–89] (23)	84 [80–89] (29)	0.9
ETDRS BSCVA, logMAR (Dunker et al. 2020)			
Preoperative	0.31 [0.26–0.37] (25)	0.37 [0.30–0.44] (29)	–*
3 months	0.22 [0.16–0.27] (24)	0.15 [0.08–0.22] (29)	0.2
6 months	0.16 [0.12–0.21] (24)	0.11 [0.05–0.17] (29)	0.2
12 months	0.15 [0.10–0.19] (24)	0.08 [0.03–0.14] (29)	0.1

BSCVA = Best spectacle‐corrected visual acuity, CB = cornea back, CF = cornea front, CI = confidence interval, DMEK = Descemet membrane endothelial keratoplasty, EMM = estimated marginal mean, ETDRS = Early Treatment Diabetic Retinopathy Study, HOAs = higher‐order aberrations, logMAR = Logarithm of the Minimum Angle of Resolution, rms = root mean square, TC = total cornea, UT‐DSAEK = ultrathin Descemet stripping automated endothelial keratoplasty, VFQ‐25 = National Eye Institute Visual Function Questionnaire 25 items.

* Not tested.

Posterior corneal HOAs did not differ between both groups before surgery. After surgery, posterior corneal HOAs were significantly lower after DMEK compared with ultrathin DSAEK at all time points, Fig. 2A. In DMEK, posterior corneal HOAs decreased at 3 months compared to baseline (*β* = −0.2 [95% CI: −0.36 to −0.4], p = 0.015) and subsequently stabilized between 3 and 12 months (*β* = −0.02 [95% CI: −0.18 to 0.14], p = 0.8). In ultrathin DSAEK, posterior corneal HOAs increased at 3 months (*β* = 0.24 [95% CI: 0.14 to 0.33], p < 0.001) and significantly decreased between 3 and 12 months (*β* = −0.21 [95% CI: −0.31 to −0.11], p < 0.001). At 3 and 12 months, in both groups, posterior corneal HOAs were significantly correlated with BSCVA, albeit weakly (*r* = 0.29, p = 0.04, and *r* = 0.29, p = 0.04; respectively), but not at baseline and 6 months (*r* = 0.22, p = 0.1, and *r* = 0.21, p = 0.2; respectively).

Total corneal HOAs did not differ significantly between DMEK and ultrathin DSAEK at all time points, Table 1, and did not change significantly over time (all *β* ≤ 0.13, all p ≥ 0.2). Total corneal HOAs significantly correlated with BSCVA at 6 months (*r* = 0.32, p = 0.023), but not at other time points.

### Contrast sensitivity

**Fig. 3:**
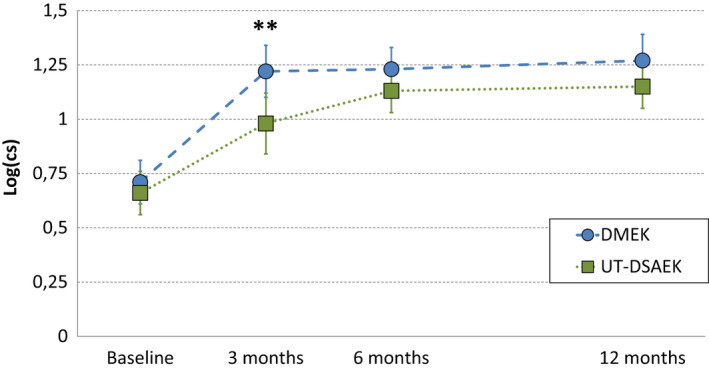
Randomized controlled trial of DMEK versus ultrathin DSAEK. Contrast sensitivity in both treatment groups. Three months after surgery, contrast sensitivity was significantly higher after DMEK compared with ultrathin DSAEK (log(cs) = 1.22 [95% CI: 1.10 to 1.35] versus log(cs) = 0.98 [95% CI: 0.84 to 1.12]; respectively, p = 0.01). Contrast sensitivity correlated negatively with best spectacle‐corrected visual acuity (logarithm of the minimum angle of resolution) at all time points (preoperative: *r* = −0.4, p = 0.003; 3 months: *r* = −0.43, p = 0.003; 6 months: *r* = −0.52, p < 0.001; 12 months: *r* = −0.53, p < 0.001). CS = contrast sensitivity value, DMEK = Descemet membrane endothelial keratoplasty, UT‐DSAEK = ultrathin Descemet stripping automated endothelial keratoplasty. **p ≤ 0.01.

Preoperative contrast sensitivity did not differ significantly between DMEK and ultrathin DSAEK. Three months after surgery, contrast sensitivity was significantly better after DMEK compared with ultrathin DSAEK but did not differ at other time points, Fig. 3. In DMEK, contrast sensitivity improved significantly at 3 months (*β* = 0.52 [95% CI: 0.37 to 0.68], p < 0.001) and stabilized thereafter (3–12 months: *β* = 0.04 [95% CI: −0.12 to 0.20], p = 0.6). In ultrathin DSAEK, contrast sensitivity improved significantly at 3 months (*β* = 0.30 [95% CI: 0.14 to 0.46], p < 0.001) and subsequently significantly improved between 3 and 12 months (*β* = 0.20 [95% CI: 0.04 to 0.36], p = 0.016). Contrast sensitivity correlated significantly with BSCVA (logMAR) at all time points (preoperative: *r* = −0.4, p = 0.003; 3 months: *r* = −0.43, p = 0.003; 6 months: *r* = −0.52, p < 0.001; 12 months: *r* = −0.53, p < 0.001).

### Straylight

**Fig. 4:**
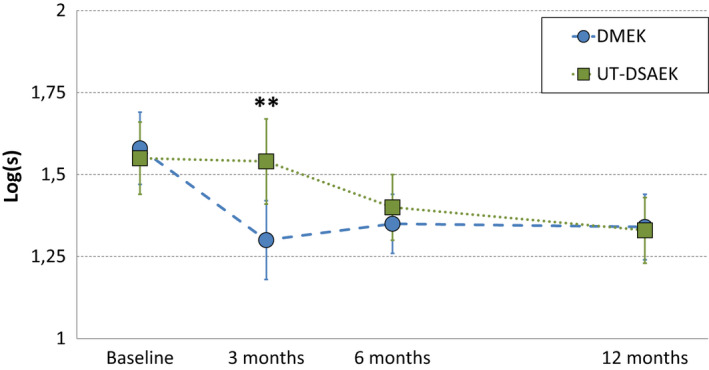
Randomized controlled trial of DMEK versus ultrathin DSAEK. Intraocular straylight in both treatment groups. Three months after surgery, straylight was lower in DMEK compared to ultrathin DSAEK (log(s) = 1.30 [95% CI: 1.18 to 1.43] versus log(s) = 1.54 [95% CI: 1.41 to 1.66], respectively, p = 0.01). In DMEK, straylight improved significantly at 3 months by *β* = 0.27 [95% CI: 0.13 to 0.41], p < 0.001, and stabilized thereafter. In ultrathin DSAEK, straylight improved significantly at 12 months compared to baseline (*β* = 0.23 [95% CI: 0.06 to 0.39, p = 0.007). DMEK = Descemet membrane endothelial keratoplasty, S = straylight value, UT‐DSAEK = ultrathin Descemet stripping automated endothelial keratoplasty. **p ≤ 0.01.

Preoperative straylight did not differ significantly between DMEK and ultrathin DSAEK. Three months after surgery, straylight was lower in DMEK compared to ultrathin DSAEK but did not differ significantly at other time points, Fig. 4. In DMEK, straylight improved significantly at 3 months by *β* = 0.27 [95% CI: 0.13 to 0.41], p < 0.001 and stabilized thereafter (3–12 months: *β* = 0.07 [95% CI: −0.07 to 0.22], p = 0.3). In ultrathin DSAEK, straylight did not change significantly at three and 6 months compared to baseline (p = 0.8 and p = 0.1, respectively), but improved significantly at 12 months compared to baseline (*β* = 0.23 [95% CI: 0.06 to 0.39, p = 0.007). Straylight did not correlate significantly with BSCVA at baseline (*r* = 0.06, p = 0.7), but correlated significantly at three and 6 months and was marginally significant at 12 months (*r* = 0.47, p = 0.003; and *r* = 0.50, p = 0.003; and *r* = 0.3, p = 0.09; respectively).

### Vision‐related quality of life

National Eye Institute (NEI )VFQ‐25 composite score and the 11 vision‐related subscales did not differ significantly between both groups at all time points, Table 1. Three months after surgery, the composite score increased significantly compared with preoperative values in both groups (*β* = 12 [95% CI: 7 to 16]; p < 0.001). Between 3 and 12 months after surgery, a subsequent marginally significant improvement in composite score was observed in both groups (*β* = 5 [95% CI: 0 to 9]; p = 0.06). In sensitivity analysis (excluding data at 6 and 12 months of patients that underwent corneal transplantation in the fellow eye during the study), mean vision‐related QOL did not differ significantly between DMEK and ultrathin DSAEK at 6 months (83 [95% CI: 79 to 87] versus 82 [95% CI: 78 to 86], p = 0.9), and 12 months (82 [95% CI: 77 to 87] versus 81 [95% CI: 76 to 87], p = 1.0).

## Discussion

In this prespecified secondary analysis of a multicentre RCT, we report quality of vision and vision‐related QOL in DMEK versus ultrathin DSAEK. Three months after surgery, posterior corneal HOAs and straylight were lower, and contrast sensitivity was higher in DMEK compared to UT‐DSAEK. Vision‐related QOL increased to a similar extend in both treatment arms.

**Fig. 5:**
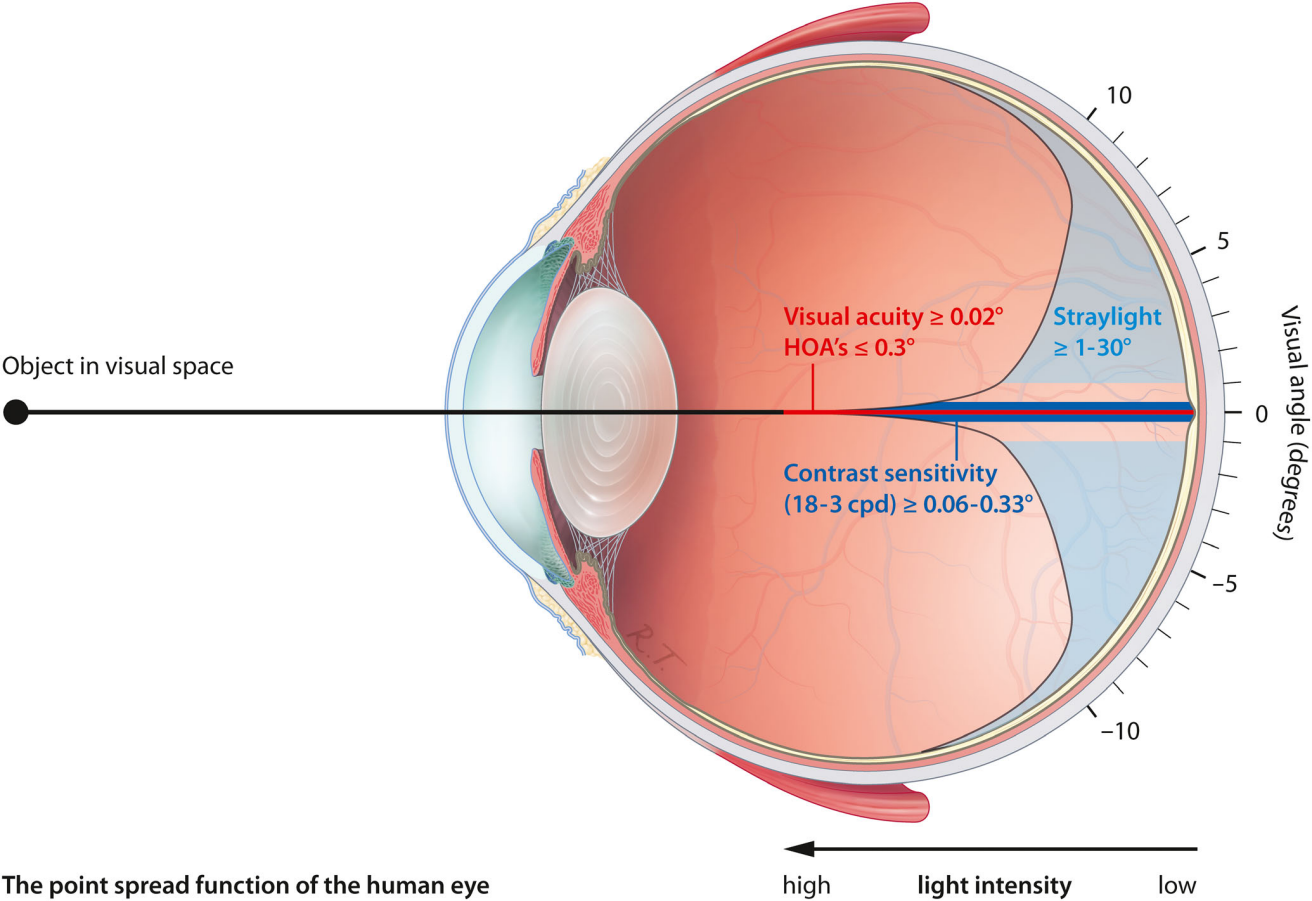
Randomized controlled trial of DMEK versus ultrathin DSAEK: Schematic retinal point spread function showing visual domains of visual acuity, higher‐order aberrations, contrast sensitivity and straylight in the human eye. The visual angle is exaggerated for clarity. Cpd = cycles per degree, DMEK = Descemet membrane endothelial keratoplasty, DSAEK = Descemet stripping automated endothelial keratoplasty, HOAs = higher‐order aberrations.

Light entering the eye and reaching the retina can be described in terms of spatial distribution and intensity. When plotted, the resulting graph is termed point spread function (see Fig. 5). High intensity is found at the very centre of the point spread function, while intensity rapidly decreases towards the periphery (van den Berg et al. 2009). When visual acuity is tested, only the very centre, an area of a few minutes of arc, is assessed. Earlier reports have shown that subjective experience of vision depends also on factors related to the large‐angle domain of the point spread function, such as contrast sensitivity and straylight (van der Meulen et al. 2012).

The majority (about 80%) of ocular aberrations occur on the corneal surface (Hamam 2003). Both anterior and posterior corneal HOAs have been suggested to impact visual acuity after EK, but reports are inconsistent (Nielsen et al. 2016; Duggan et al. 2019). In the current study, anterior corneal HOAs increased postoperatively in both treatment arms, stabilizing after 3 months. The increase in anterior corneal HOAs is likely related to surgical incisions, wound healing, and subepithelial fibrosis (Patel et al. 2012).

Anterior corneal HOAs did not differ significantly between ultrathin DSAEK and DMEK. Posterior corneal HOAs decreased after DMEK and increased 3 months after ultrathin DSAEK, decreasing subsequently at 6 and 12 months. Graft asymmetry (Dickman et al. 2013), graft folds (Seery et al. 2011) and a donor‐recipient curvature mismatch (Yamaguchi et al. 2015) may be responsible for the increase in posterior corneal HOAs after ultrathin DSAEK. Interestingly, compared to anterior and total corneal HOAs, posterior corneal HOAs showed the strongest correlation with BSCVA. Nevertheless, the correlation was only weak to modest (*r* ≤ 0.29). This is likely owing to the smaller change in refractive index at the posterior cornea compared with that at the anterior cornea.

To the best of our knowledge, this is the first study to directly compare contrast sensitivity and straylight in DMEK versus ultrathin DSAEK. In both treatment arms, preoperative contrast sensitivity and straylight values were worse compared to healthy age‐matched eyes (Hashemi et al. 2012; Labuz et al. 2015). This may be attributed to structural changes in the recipient’s Descemet membrane and corneal oedema (Watanabe et al. 2015). After surgery, patients in both treatment arms achieved near‐normative values of contrast sensitivity and straylight 3 months after DMEK and 12 months after ultrathin DSAEK. In straylight, a decrease of 0.1 log(s) has approximately the same impact as a gain of 0.1 logMAR and may therefore be considered clinically relevant (Labuz et al. 2015). Three months after surgery, a clinically relevant mean difference of 0.24 log(s) was observed in favour of DMEK. One year after surgery, straylight improved by approximately 70% with both techniques compared with baseline. A doubling in contrast sensitivity may be considered clinically relevant (Legge et al. 1987). Three months after surgery, a mean difference of 0.24 log(CS) was observed in favour of DMEK, nearly equivalent to twice better contrast sensitivity. One year after surgery, contrast sensitivity tripled with both techniques compared to baseline. In ultrathin DSAEK, slower recovery may be due to light scattering at the stroma‐to‐stroma interface, or from the added tissue itself, as even normally hydrated corneal stroma scatters light (Olsen 1982).

In clinical research, identifying outcomes relevant to patients is vital (Seligman et al. 2019). Benefit, as perceived by patients, is not necessarily revealed by clinical outcome measures and may vary between individuals with similar objective outcomes. This subjective dimension can be captured by patient reported outcome measures. Vision‐related QOL did not differ between DMEK and ultrathin DSAEK at all time points. Preoperatively, vision‐related QOL was lower in both groups compared to age‐matched controls (Mangione et al. 2001). Three months after surgery, the composite score of the VFQ‐25 increased by 12 points, followed by a small and marginally significant improvement at 12 months. This overall improvement is considered clinically relevant based on a study suggesting a cut‐off value of 10 points (Lindblad & Clemons 2005). Although a higher percentage of DMEK eyes reached 0.8 Snellen BSCVA (Dunker et al. 2020), and recovery of contrast sensitivity and straylight was faster, this did not translate to better vision‐related QOL. The DETEC trial made a similar observation comparing DMEK to ultrathin DSAEK using the same questionnaire (Ang et al. 2019). Twelve months after surgery, the composite scores of both groups remained lower compared to healthy eyes (Mangione et al. 2001), indicating incomplete recovery. However, corneal remodelling is a continuous process and vision‐related QOL has been reported to improve up to 3 years after surgery (Trousdale et al. 2014). Corneal transplantation of the fellow eye may impact outcomes of vision‐related QOL. To reduce bias from fellow eye surgery on vision‐related QOL, we performed a sensitivity analysis excluding data at 6 and 12 months of patients that underwent corneal transplantation in the fellow eye during the study. Importantly, our findings remain unchanged.

As the difference in visual acuity between the latest iterations of EK grows smaller, comprehensive evaluation of visual function and vision‐related QOL becomes increasingly important. This RCT provides the most comprehensive evaluation of visual function to date after DMEK and ultrathin DSAEK. Interestingly, better objective outcomes after DMEK did not translate to higher vision‐related QOL in the current study. One possible explanation may be that the NEI VFQ‐25 lacks sensitivity to capture relevant domains after corneal transplantation. Two questionnaires have recently been validated to measure subjective visual function in corneal transplantation, Catquest‐9SF (Claesson et al. 2017), and specifically for FECD, V‐Fuchs (Wacker et al. 2018). V‐Fuchs incorporates disease‐specific domains, such as glare disability and diurnal shift, but is also more extensive and only validated in English. For future clinical research on treatments for corneal disease, both questionnaires are promising. Another limitation of this secondary analysis pertains to the statistical power. Sample size was based on the expected difference in primary outcome, that is BSCVA (Dunker et al. 2020). Therefore, statistical power may be insufficient for the parameters assessed in the current study. However, this study did not identify clinically relevant differences that merely failed to reach statistical significance, suggesting that a bigger sample size would lead to smaller confidence intervals without materially altering our conclusions.

In our cohort, we observed no rejection episodes in both treatment arms (Dunker et al. 2020). Although encouraging, our study was insufficiently powered to assess this adverse event. The reported risk of immune rejection is approximately 10% after DS(A)EK and 2% after DMEK (Deng et al. 2018). Taken together, the results of both RCTs comparing DMEK and ultrathin DSAEK and the prospective study by Busin et al. suggest the 1‐year rate of immune rejection after ultrathin DSAEK (0–3.4%) is closer to DMEK than DSAEK (Madi et al. 2019).

In the primary report of this RCT, we showed that BSCVA did not differ significantly between DMEK and UT‐DSAEK but a significantly higher percentage of eyes reached 20/25 Snellen after DMEK (Dunker et al. 2020). In this prespecified sub‐analysis of the same cohort, DMEK showed faster recovery of straylight and contrast sensitivity and lower posterior corneal HOAs compared to ultrathin DSAEK. Posterior corneal HOAs correlated weakly to moderately with BSCVA. Vision‐related QOL improved significantly in both groups to a similar extend.
